# Endoscope-assisted resection of cavernous angioma at the foramen of Monro: a case report

**DOI:** 10.1186/s40064-016-3538-x

**Published:** 2016-10-20

**Authors:** Yuji Matsumoto, Kazuhiko Kurozumi, Yousuke Shimazu, Tomotsugu Ichikawa, Isao Date

**Affiliations:** 1 Department of Neurological Surgery, Okayama University Graduate School of Medicine, Dentistry and Pharmaceutical Sciences, 2-5-1 Shikata-cho, Okayama, Okayama 700-8558 Japan; 2 Department of Neurological Surgery, Hiroshima City Hiroshima Citizens Hospital, Hiroshima, Japan

**Keywords:** Cavernous angioma, Foramen of Monro, Neuroendoscope-assisted surgery

## Abstract

**Introduction:**

Intraventricular cavernous angiomas are rare pathological entities, and those located at the foramen of Monro are even rarer. We herein present a case of cavernous angioma at the foramen of Monro that was successfully treated by neuroendoscope-assisted surgical removal, and review the relevant literature.

**Case presentation:**

A 65-year-old woman had experienced headache and vomiting for 10 days before admission to another hospital. Magnetic resonance imaging (MRI) showed a mass at the foramen of Monro, and obstructive hydrocephalus of both lateral ventricles. The patient was then referred to our hospital. Neurological examination on admission to our hospital showed memory disturbance (Mini-Mental State Examination 20/30) and wide-based gait. A cavernous angioma at the foramen of Monro was diagnosed based on the typical popcorn-like appearance of the lesion on MRI. The lesion was completely removed by neuroendoscope-assisted transcortical surgery with the Viewsite Brain Access System (Vycor Medical Inc., Boca Raton, FL), leading to a reduction in the size of the ventricles. The resected mass was histologically confirmed to be cavernous angioma. The patient’s symptoms resolved immediately and there were no postoperative complications.

**Conclusion:**

Minimally invasive neuroendoscope-assisted surgery was used to successfully treat a cavernous angioma at the foramen of Monro.

## Background

Intraventricular cavernous angiomas are rare pathological entities, constituting 2.5–10.8 % of cerebral cavernous angiomas (Kivelev et al. 2010); those localized at the foramen of Monro are even rarer. To the best of our knowledge, only 16 cases of cavernous angioma at the foramen of Monro have been previously reported (Lee et al. 2012; Bhatia et al. 2013; Winslow et al. 2015). Surgical removal was performed in all previous cases. Most removals were performed via microsurgery; however, neuroendoscopic surgery is being used increasingly more frequently. We herein describe a recent case of a cavernous angioma at the foramen of Monro that was successfully treated using neuroendoscope-assisted surgery.

## Case presentation

A 65-year-old woman with a history of hyperlipidemia had experienced headache and vomiting for 10 days before admission to another hospital. Magnetic resonance imaging (MRI) showed enlargement of both lateral ventricles and a mass at the foramen of Monro. The patient was then referred to our hospital.

**Fig. 1:**
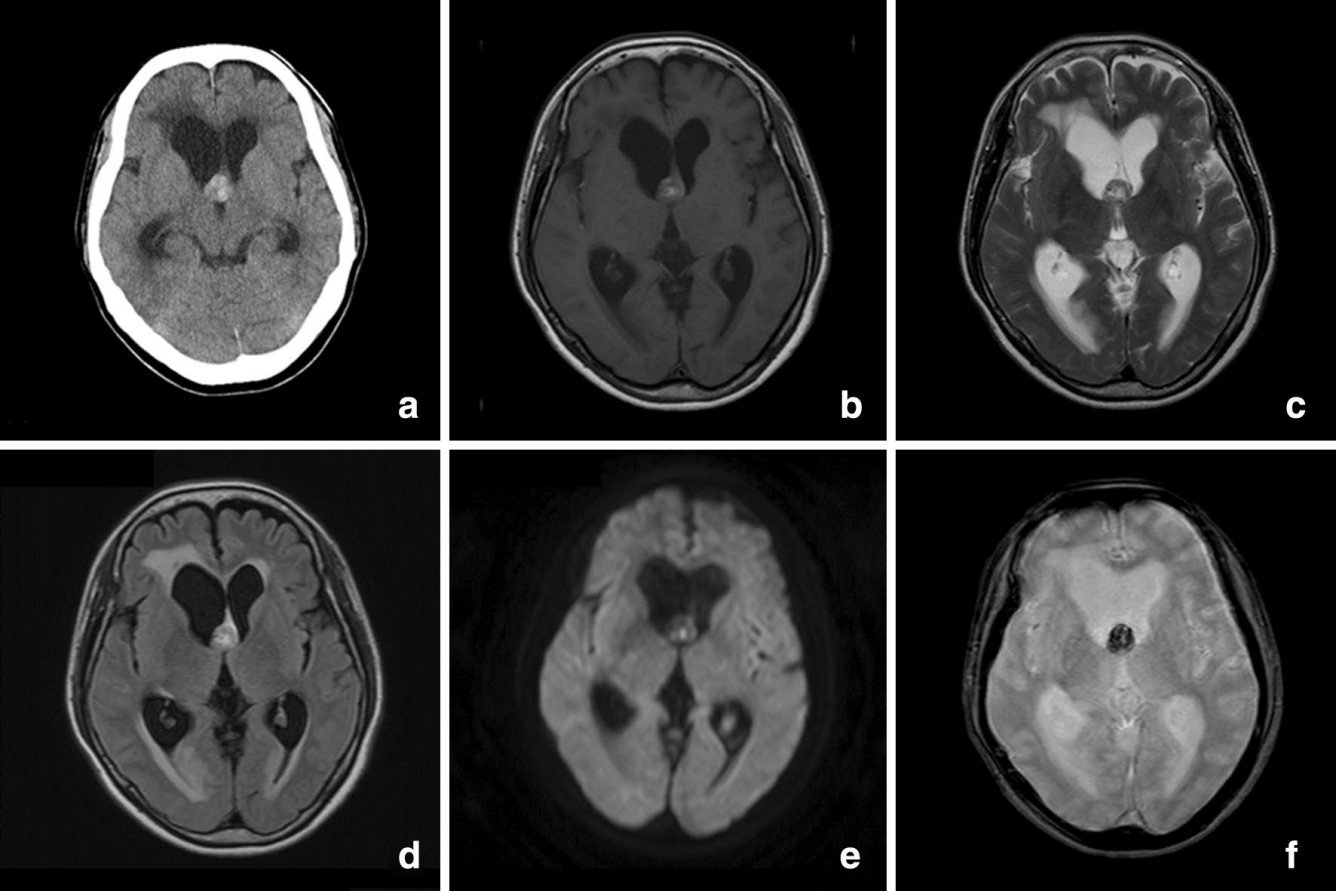
Computed tomography and magnetic resonance imagings. **a** There was a mild hyperintense 16-mm-diameter mass without calcification at the foramen of Monro causing obstructive hydrocephalus. **b**, **c** T1- and T2-weighted images showed the well-delineated mixed-signal heterogeneous core. The typical peripheral hemosiderin rim of low signal intensity was not seen on T2-weighted imaging. **d** No perilesional edema was presented on the fluid-attenuated inversion recovery magnetic resonance image. **e** Diffusion-weighted imaging showed an isointense mass; only a portion of the mass was hyperintense. **f** T2-star-weighted imaging showed a hypointense mass

**Fig. 2:**
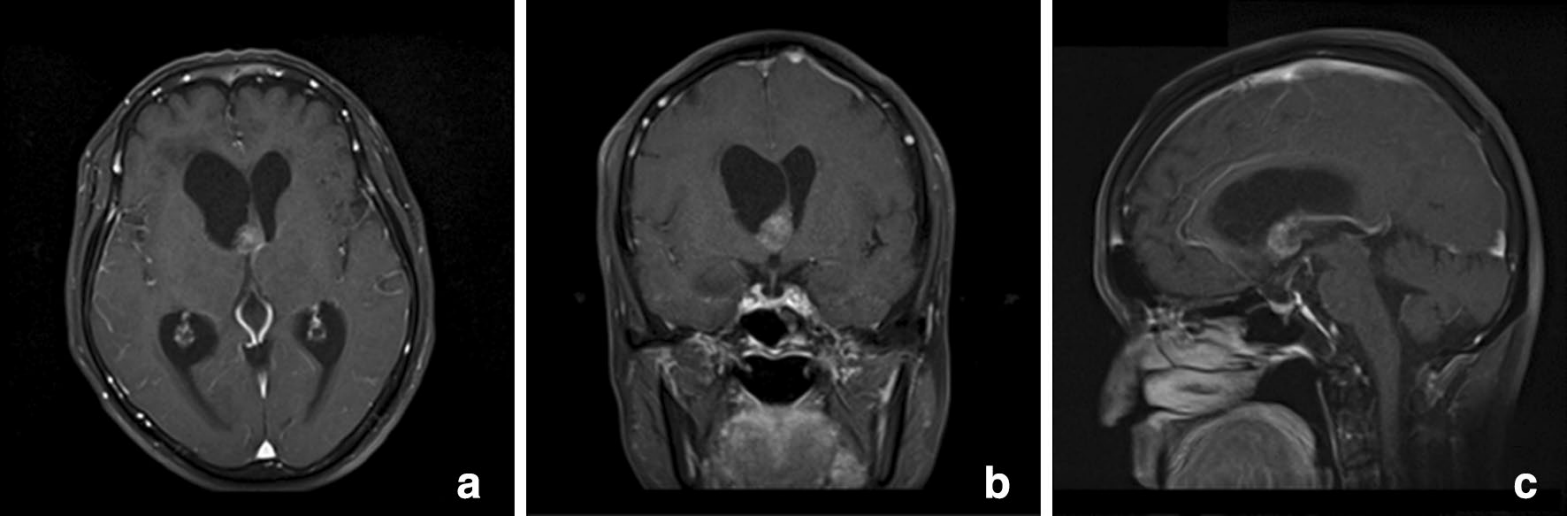
**a** Axial, **b** coronal, and **c** sagittal gadolinium-enhanced T1-weighted imaging demonstrated mild mass enhancement

Neurological examination on admission to our hospital showed memory disturbance (Mini-Mental State Examination 20/30) and a wide-based gait disturbance. Computed tomography (CT) showed a 16 mm mildly hyperdense mass with no calcification at the foramen of Monro, and the mass was causing obstructive hydrocephalus. MRI revealed a well-delineated mass at the foramen of Monro with heterogeneous signal intensity on both T1- and T2-weighted images; the mixed-signal core appeared as a popcorn-like lesion typical of cavernous angioma (Fig. 1). Gadolinium-enhanced T1-weighted imaging revealed mild enhancement of the mass (Fig. 2). There were no vascular abnormalities on CT angiography or CT venography. We considered a colloid cyst, central neurocytoma, subependymoma, ependymoma, low grade astrocytoma, and arteriovenous malformation as differential diagnosis; however, despite the unusual location of the mass, a diagnosis of intraventricular cavernous angioma was made because of its typical appearance on MRI.

**Fig. 3:**
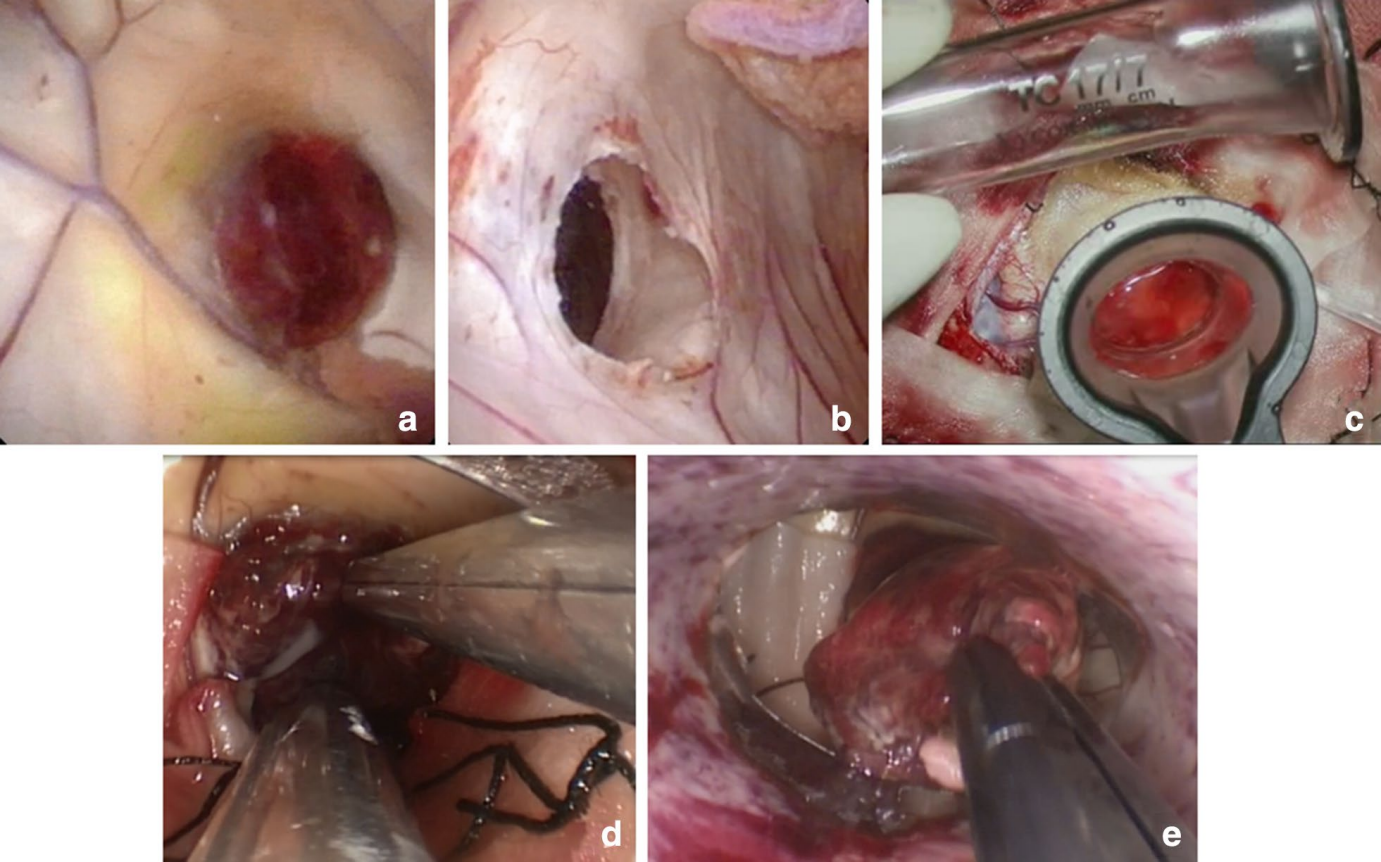
Intraoperative photographs of the mass resection procedure. **a** Intraoperative neuroendoscopy image showing the *reddish* lobular mass with hematoma and obstruction of the foramen of Monro. **b** Cavum septum pellucidum had already occurred because of the high intracranial pressure associated with hydrocephalus. **c** Insertion of clear plastic sheath (ViewSite) into the brain. **d** The mass was bluntly dissected from the ventricle wall by one surgeon. **e** Total en bloc resection of the mass was performed

We performed endoscope-assisted transcortical removal of the mass (Fig. 3). The entry point was made using the StealthStation S7 navigation system (Medtronic Inc., Louisville, CO), and a flexible videoscope (VEF-V, Olympus Corporation, Tokyo, Japan) was inserted. Intraoperative neuroendoscopic imaging revealed a reddish lobular mass with a hematoma and obstruction of the foramen of Monro. We observed the cavum septum pellucidum because of the high intracranial pressure associated with hydrocephalus. After right frontal mini-craniotomy, the Viewsite Brain Access System (Vycor Medical Inc., Boca Raton, FL) was inserted (Raza et al. 2011); we used the 17 mm wide retractor in the 7 cm length. Endoscope-assisted surgery with the Viewsite was performed with technique similar to microsurgery. A 2.7 mm rigid endoscope (Karl Storz, Tuttlingen, Germany) fixed by UniArm (Mitaka Kohki, Tokyo, Japan) was inserted. The working ambience was air because of its advantages over fluid ambience especially when dealing with a relatively vascularized pathology. Other microsurgical instruments were used parallel to the endoscope. The endoscope served only as an optic apparatus. We used the Viewsite as an access port to enable dual instrumentation (endoscope and microsurgical instrumentation). The tumor was bluntly dissected from the ventricle wall, and total en bloc resection of the lesion was performed by one surgeon using the two-handed technique. Bleeding was well controlled with irrigation and bipolar coagulation.

**Fig. 4:**
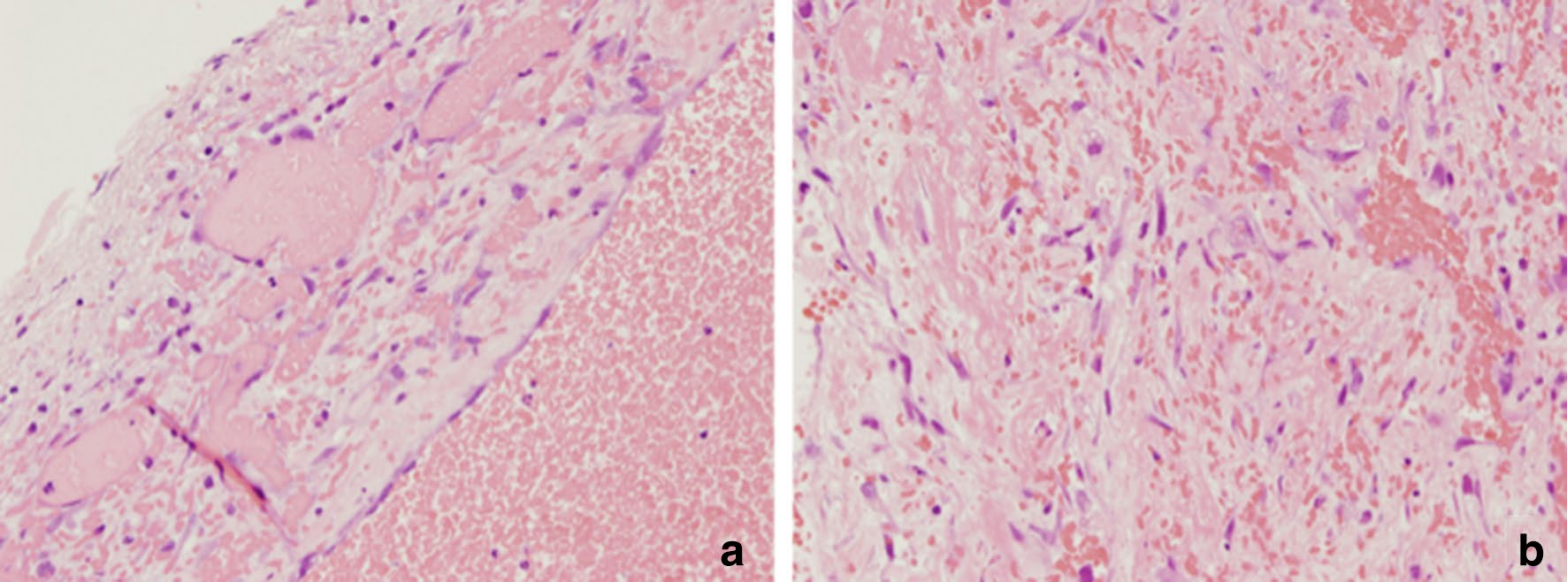
Photomicrograph of the lesion showing large vascular spaces filled with an organized thrombus Specimens were stained with hematoxylin and eosin. **a** ×100 magnification. **b** ×400 magnification

The resected tumor was reddish and consisted mainly of clotted blood vessels and xanthochromic tissue. Histological examination revealed a cavernous angioma with evidence of large vascular spaces filled with an organized thrombus (Fig. 4).

**Fig. 5:**
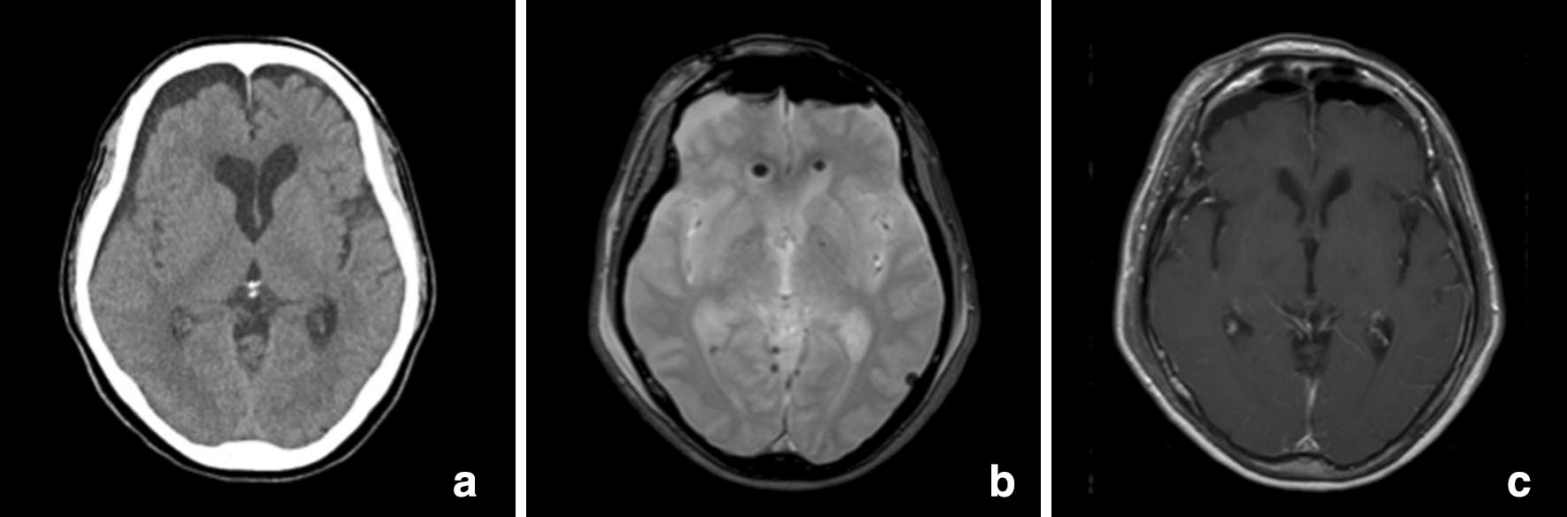
Postoperative imaging showed no evidence of residual mass, and demonstrated improvement of hydrocephalus. **a** Postoperative computed tomography image, **b** T2-star-weighted image, and **c** gadolinium-enhanced T1-weighted image

Postoperative MRI confirmed complete removal of the tumor, and a return to normal ventricular size (Fig. 5). The patient’s symptoms resolved immediately, and there were no postoperative complications. She was discharged without any neurological deficit. No complications or neurological impairment were observed at the 1-year follow-up.

## Discussion

The details of all 17 cases of cavernous angioma located at the foramen of Monro reported to date, including our case, are summarized in Table 1. The patients comprised 5 males and 12 females, with a mean age of 44 years (range 11–65 years). Hydrocephalus occurred in all but one patient; this was due to obstruction of the cerebrospinal fluid (CSF) pathway in 13 patients, and acute hemorrhage in 3 patients. Frequent clinical findings were headache, vomiting, ataxia, memory disturbance and disorientation, all of which were associated with hydrocephalus in most patients. In our case, headache, vomiting, ataxia and memory disturbance resulted from increased intracranial pressure due to obstructive hydrocephalus induced by the cavernous angioma at the foramen of Monro.

**Table 1:** Summary of the 17 reported cases of cavernous angioma at the foramen of Monro

Author, year	Age (years)/sex	Symptom	Surgical approach/side	Microscopy or endoscopy	Outcome
Britt et al. (1980)	11/F	Nausea, vomit	Transcortical/right	Microscopy	No deficit
Pozzati et al. (1981)	31/F	Nausea, vomit	Transcortical/right	Microscopy	No deficit
Harbaugh et al. (1984)	44/F	Headache, nausea, meningismus,hemorrhage	Transcallosal	Microscopy	Hydrocephalus and partially amnesia
Katayama et al. (1994)	50/F	NA	Transcallosal	Microscopy	No deficit
Katayama et al. (1994)	45/F	Massive hemorrhage	Transcortical	Microscopy	Vegetable state
Kaim et al. (1997)	64/M	Headache, tinnitus, ataxia, memory disturbance	Transcallosal	Microscopy	No deficit
Crivelli et al. (2002)	38/M	Short term memory loss, headache, vomit, nausea, ataxia, disorientation	Transcortical/left	Microscopy	No deficit
Suess et al. (2002)	36/F	Short term memoly loss	Transcallosal	Microscopy	No deficit
Chen et al. (2006)	51/F	Headache, ataxia, vomit, conscious change, disorientation	Transcortical	Microscopy	No deficit
Longatti et al. (2006)	35/M	Headache, vomit	Transcallosal	Endoscope-assisted	No deficit
Sato et al. (2006)	47/F	Headache	Transcallosal/right	Microscopy	No deficit
Prat et al. (2008)	56/M	Headache, confusion, hemorrhage,	Transcortical/left	Endoscopy	No deficit
Kivelev et al. (2010)	52/M	Headache, nausea, vomit	Transcallosal	Microscopy	No deficit
Lee et al. (2012)	30/F	Headache, short term memory loss, vomit	Transcallosal	Microscopy	No deficit
Bhatia et al. (2013)	29/F	Headache, vomit	Transventricular	Endoscopy	NA
Winslow et al. (2015)	64/F	Unresponsiveness	Ventriculostomy	none	death
Present case	65/F	Headache, vomit, gait disturbance, memory disturbance	Transcortical/right	Endoscope-assisted	No deficit

*NA* not available

Surgical removal was performed in all patients. Microsurgery was performed in 12 patients (transcallosal approach in 6, transcortical approach in 6), endoscope-assisted surgery in 2 patients (transcallosal approach in 1, transcortical approach in 1), and endoscopic surgery 2 patients (transcortical approach in 1, transventricular approach in 1).

Thirteen patients demonstrated full recovery of neurological function after surgery. Of the other four patients, one retained mild neurological deficits due to postoperative hydrocephalus, one fell into a persistent vegetative state due to massive hemorrhage at symptom onset, one who was found unresponsive with decorticate posturing to noxious stimuli died, and one was not available for follow-up.

### Surgery for cavernous angioma

The treatment goal in patients with cavernous angioma is total removal because postoperative remnants increase the risk of regrowth and bleeding. Intraventricular tumors and cysts are ideal lesions for the application of neuroendoscopy; good visualization is possible because of their location inside the CSF-filled ventricular system, and the often-associated obstruction of the CSF pathway and ventricular enlargement offer the possibility of working in large spaces (Cappabianca et al. 2008).

### Endoscope-assisted resection

The ideal tumors for endoscopic surgery should exhibit the following characteristics: moderate vascularity, soft consistency, small diameter (2–3 cm), associated hydrocephalus, and low histological grade (Cappabianca et al. 2008). The microsurgical approach may ensure a higher level of precision in patients with highly vascularized tumors (glioblastoma with arteriovenous shunt, solid hemangioblastoma), relatively solid tumors, tumors with firm adhesion to surrounding tissue, and tumors in which the approach route crosses important structures (Kishida et al. 2014). However, in selected cases, the endoscopic approach to intraventricular and paraventricular tumors is less invasive and similarly effective compared with microsurgical resection (Cappabianca et al. 2008).

The endoscopic approach is categorized into endoscopic resection and endoscope-assisted resection. The main differences between these two techniques are whether the working ambience is water or air and whether transendoscopic instrumentation is used or other microsurgical instruments are used in parallel with the endoscope (in such cases, the endoscope serves only as an optic apparatus). In the present case, we chose endoscope-assisted resection because we could use the Viewsite as an access port to enable dual instrumentation (endoscope and microsurgical instrumentation) and work in an air ambience. We performed bimanual dissection by freeing both hands through a smaller skin incision, craniotomy, and corticotomy and inducing minimal white matter damage (Kishida et al. 2014). We resected the tumor in a much safer and more effective manner than conventional purely endoscopic resection (Kishida et al. 2014).

The main advantage of the endoscope-assisted approach is that it is less invasive because it induces less white matter damage than does the microscopic approach. The tubular retractors require a larger-diameter conduit to allow for bimanual operation of the microscope because the microscope is used to deliver a cone of light as it progressively tapers from the source to the target. However, the endoscope affords a panoramic view via an inverted cone of light (“flashlight effect”) and allows dynamic magnification (McLaughlin et al. 2013).

### Usefulness of Viewsite and electromagnetic navigation

The Viewsite (Vycor Medical Inc.) is a tubular retractor system designed specifically for intracranial use. It consists of an introducer that permits entry into the tissue and a working channel, and it has transparent plastic walls that permit visualization of surrounding tissue (Raza et al. 2011). The Viewsite is available in four widths: 12, 17, 21, and 28 mm; it is also available in three lengths: 3, 5, and 7 cm. We primarily use the 12 or 17 mm wide retractor in either the 5 or 7 cm length. Endoscopic assisted surgery with the Viewsite is conducted using a technique similar to that used in microsurgery.

The use of neuronavigation in preoperative trajectory planning and establishment of intraoperative landmarks to avoid morbidity was helpful when the lesion overlapped the fornix and feeding vessels of the choroid plexus (Prat and Galeano 2008). Whenever available, intraoperative stereotactic navigation should be considered for all cases of endoscopic resection of cavernous angioma at the foramen of Monro. If intraoperative neuronavigation is not available, the entry point should be estimated from the preoperative imaging studies. An estimated entry point of 4.0 cm perpendicular to the midline and 4.5 cm anterior to the coronal suture is an acceptable alternative that can be used in patients with ventriculomegaly (Rangel-Castilla et al. 2014). Current navigation systems use either optical or electromagnetic tracking. In neuroendoscopic surgery, electromagnetic technology is more useful because it avoids the “line-of-sight” problem often encountered in optical navigation systems.

## Conclusion

We experienced an extremely rare case of cavernous angioma at the foramen of Monro. Minimally invasive neuroendoscope-assisted surgery was used to successfully remove the tumor without complications.

## Abbreviations

CSF: cerebrospinal fluid
CT: computed tomography
MRI: magnetic resonance imaging
